# Curriculum in Pharmacoepidemiology Training Programs: A Cross‐Sectional Study to Assess Educational Needs and Alignment With Core Competencies

**DOI:** 10.1002/pds.70351

**Published:** 2026-03-26

**Authors:** Amie Goodin, Deborah Layton, Ryan Chung, Vicki Osborne, Chioma Ejekam, Xiaojuan Li, Xuerong Wen, Kristian B. Filion, Luciane Cruz Lopes, Daniela C. Moga

**Affiliations:** ^1^ Department of Pharmaceutical Outcomes and Policy University of Florida Gainesville Florida USA; ^2^ PEPI Consultancy Limited Southampton UK and University of Hertfordshire Hatfield UK; ^3^ Lane, Clark and Peacock, LLP London UK; ^4^ Jazz Pharmaceuticals Cambridge UK; ^5^ ACEDHARS University of Lagos Lagos Nigeria; ^6^ Harvard Medical School Department of Population Medicine and Harvard Pilgrim Health Care Institute Boston Massachusetts USA; ^7^ University of Rhode Island Kingston Rhode Island USA; ^8^ Departments of Medicine and of Epidemiology, Biostatistics, and Occupational Health McGill University Montreal Canada; ^9^ Pharmaceutical Science Graduate Course University of Sorocaba São Paulo Brazil; ^10^ Department of Pharmacy Practice and Science, College of Pharmacy and Department of Epidemiology and Environmental Health, College of Public Health University of Kentucky Lexington Kentucky USA

**Keywords:** core competencies, curriculum assessment, educational needs assessment, pharmacoepidemiology curriculum, pharmacoepidemiology education, pharmacoepidemiology training

## Abstract

**Purpose:**

Pharmacoepidemiology is a key discipline for evidence‐based decision‐making, yet its educational programs have not been systematically assessed for alignment with international core competencies. Our objectives were to evaluate curricula alignment of pharmacoepidemiology training programs with ISPE‐recommended core competency themes and identify key curriculum content development areas.

**Methods:**

The ISPE Core Competencies Workgroup and ISPE leadership developed a curriculum and needs assessment survey, incorporating feedback from global members. The survey was electronically distributed to leaders of pharmacoepidemiology training programs (e.g., department chairs and program directors) using the ISPE contact database, followed by snowball sampling to enhance representation. Institutional respondent characteristics were categorized by sector and geographic region. Competency mapping identified the most and least represented competency categories within curricula. Needs assessment responses were reported as proportions, and free‐text responses were thematically analyzed, allowing for multiple themes per response.

**Results:**

Sixty‐four institutions participated (73.4% academic programs, 18.8% industry/consulting, and 7.8% government/regulatory). Representation by global region was: 35.9% Europe, 29.7% North America, 23.4% Latin America, 7.8% Asia, 1.6% Africa, and 1.6% Gulf Region. Respondents prioritized developing standards in pharmacoepidemiology training curriculum in two areas—45.5% “Epidemiology” and 36.4% “Statistics, Analysis, and Data Science”—with few endorsements of other areas. The most represented competencies covered in curricula were “Statistics” (100%, 45/45) and “Study Design” (100%, 43/43). The least represented were “Advanced Modeling” (54%, 25/46) and “Professional Practice” (66%, 31/47). Key needs in pharmacoepidemiology curriculum centered around four themes: 33.3% (8/24) “Methods (generally)”, 33.3% (8/24) “Analytical Skills,” 16.7% (4/24) “Communication (writing, teaching),” and 25.0% “(6/24) Other (applications, new therapies).” Content type that would best support curriculum development, as ranked by 42 respondents, was: “Learning objectives with pre‐packaged activities” 45.2% (19/42), “Recorded webinars/lectures” 28.6% (12/42), “Example syllabi” 21.4% (9/42), and “Reading lists” 4.8% (2/42).

**Conclusions:**

This global assessment highlights critical gaps in pharmacoepidemiology training, particularly in advanced analytical methods and professional practice. ISPE has a unique opportunity to address these gaps by developing targeted educational activities and resources that enhance methodological rigor and practical skills.

## Plain Language Summary

Pharmacoepidemiology is a field of study that has changed due to improvements in technology and a better understanding of tools to use in research. No one yet knows whether pharmacoepidemiology programs across the globe are covering similar topics and teaching similar skills. Our goals for this study were to learn more about what these programs around the world teach and what program leaders say that they need to help their students learn pharmacoepidemiology skills. We surveyed 64 program leaders from around the world and found that most programs were training students in areas related to statistics and study design, but few programs were teaching students about communication skills, such as writing and teaching, professional practice, and an advanced type of research and analysis skill known as modeling. Most program leaders say that they need educational resources that combine learning objectives, or goals for learning, with hands‐on activities to help their students learn pharmacoepidemiology skills.

## Key Points

- Core competencies in pharmacoepidemiology were updated in 2024.
- This study surveyed all known pharmacoepidemiology training programs for curriculum alignment with revised core competencies, and to identify educational needs.
- From 64 participating programs around the world, most programs cover competencies related to statistics and study design in their curriculum, while few programs cover competencies related to communication, advanced modeling, and professional practice.
- More program representatives preferred educational content type that included learning objectives with pre‐packaged activities than other content types.
- ISPE is uniquely positioned to address the noted educational gaps and has several ongoing and developing initiatives in support of improving pharmacoepidemiology training in core competencies.

## 1 Introduction

The definition of pharmacoepidemiology as endorsed by the International Society for Pharmacoepidemiology (ISPE) was recently updated with an expanded scope. This revised definition, now describing the field as a “… scientific discipline that uses epidemiological methods to evaluate the use, benefits and risks of medical products and interventions in human populations,” [1] suggests an opportunity for renewed consideration by pharmacoepidemiology training programs as to the scope and depth of their curriculum. The first paper to specify the core content of pharmacoepidemiology as a profession was published by an ISPE workgroup in 2012 [2]. The field of pharmacoepidemiology has evolved since that time as new therapies necessitated different research approaches, new analytic techniques, and study designs have been developed, and technological advances allow for improved accessibility of data.

To better understand the training and educational needs in this era of matured real‐world evidence (RWE) and increased analytic tool availability, an ISPE workgroup recently published an update to the pharmacoepidemiology core competencies. The updated core competencies better reflect the training and educational needs of pharmacoepidemiologists acting within academic research environments, regulatory agencies, service providers, and industry, as they are shaped by the increasing role of RWE in decision‐making. The updated core competencies include a broader coverage of sophisticated study designs and analytical techniques used when working with real‐world data and in the generation of RWE [3].

As an international professional organization, ISPE is “… dedicated to advancing the health of the public by providing a global forum for the open exchange of scientific information and the development of policy, education, and advocacy for the field of pharmacoepidemiology,” [4] and as such, is positioned as a leader in pharmacoepidemiology education and training. The society endorses and hosts a variety of resources and content available to members; however, to date there has been no systematic examination of curricula covered within pharmacoepidemiology training programs worldwide to identify educational needs and gaps. The objectives of this project are therefore to: (1) evaluate curricula alignment of pharmacoepidemiology training programs with ISPE‐recommended core competency themes, and (2) identify key curriculum content development areas that can inform ISPE strategic planning related to training and education initiatives.

## 2 Methods

### 2.1 Study Design and Instrument Development

The curriculum and needs assessment was conducted as a cross‐sectional survey. The initial draft of the survey instrument was developed by the workgroup members that updated pharmacoepidemiology core competencies, following ISPE Academic Council request to surveil pharmacoepidemiology curriculum and identify educational and training needs in the context of the updated core competencies. Most workgroup members participated in the initial draft phase, which was accomplished through a brainstorming session to draft survey items during an online meeting. The draft survey items were then circulated via email to the remaining workgroup members who were not present at the initial survey item draft meeting with a request to provide feedback on draft item language and recommendations to add or remove draft items. Contributions to either the initial survey item drafting or through providing feedback on the draft survey items were made by all original workgroup members (*n* = 15 members).

Workgroup member qualifications and expertise are described in detail elsewhere [3]; in brief, members participating in the instrument development phase included a range of experience, from early career through advanced experience, and members represented regulatory agencies or health authorities, industry (pharmaceutical as well as contract research organizations), academic institutions, independent consultants, and healthcare providers in clinical practice. Workgroup members included representatives from several countries across four continents, and several were members of the ISPE Academic Council or ISPE leadership. Education and training experience of workgroup members varied widely, with several members maintaining faculty positions and having experience in developing pharmacoepidemiology courses, including ISPE‐endorsed or provided educational content (e.g., ISPE short courses). Feedback on draft survey items was reviewed by the full workgroup via email and in a follow‐up meeting, where revisions were made to the instrument in response to comments requesting language clarification and removal or addition of items. Survey items on the draft instrument included characteristics of the training program (e.g., sector, geographic location, type of training offered, degree‐granting status), alignment of the respondent program's current curriculum with the updated core competencies, perceived curriculum gaps and needs, or recommendations based on the updated core competencies.

### 2.2 Instrument Testing and ISPE Member Inclusion

Workgroup members then invited members of the ISPE Academic Council to contribute to testing the instrument and to assist with ISPE member outreach. The curriculum and educational needs assessment survey instrument was tested via a pilot distribution with request for feedback using a strategy akin to a simplified Delphi process [5]. Specifically, the instrument draft was distributed to attendees of a workshop included in the 2023 annual ISPE meeting following the introduction and presentation of the revised core competencies to ISPE members. The workshop, titled “Curriculum in Pharmacoepidemiology Training and Alignment with Core Competencies,” organized by workgroup leaders, contained break‐out session and small‐group discussion‐based activities. General ISPE members in attendance for these activities were requested to provide written feedback on survey items as well as recommendations for improving outreach strategies to pharmacoepidemiology training programs that were not represented at the time in the ISPE Program Directory. ISPE members contributing to this pilot testing and instrument review are characterized as a convenience sample based on workshop participation; the sample had representation from all sectors actively involved in the society (i.e., academic institutions, government or regulatory agencies, industry, and clinical practice). Final revisions were made to the survey instrument by project leaders in accordance with pilot testing feedback from ISPE members. Consensus on the final survey instrument was achieved when workgroup members in attendance at the follow‐up meeting agreed to proceed with survey distribution.

The needs assessment survey instrument was finalized and circulated to programs prior to the publication of the updated core competencies; therefore, there were minor discrepancies between the competencies included in this survey and those in the final published list. Specifically, two of the individual competencies appearing in the final updated core competencies manuscript did not appear in this survey (“Evaluation of effectiveness and impact” and “Understanding of biological mechanisms”) as these were added to the core competencies in the final stages of updates. One competency (“Double robustness”) is included in this survey assessment, but this competency does not appear in the final list of 55 individual updated core competencies as it was removed prior to publication following workgroup discussion and re‐review. The final survey instrument is included as Supporting Information: Appendices 1 and 2 includes a list of ISPE‐endorsed core competencies and their alignment with the individual core competencies within the survey instrument.

### 2.3 Data Collection

Data collection involved the use of a census strategy, rather than a sample survey, as the group attempted to collect responses from all known pharmacoepidemiology training programs that are represented in ISPE, as documented on the ISPE program contact information online directory as of August 2023. The survey instrument was made available electronically through a shareable URL, where the link directed the respondent to the instrument as hosted on Qualtrics. The instrument was accessible through a web browser, with further accessibility available via mobile phone enabled display. All ISPE educational and other training program contacts listed in the online directory of the ISPE website in August 2023 were sent a personal email invitation containing the URL and explanation of this initiative. ISPE members representing a program must opt in to the directory for educational programs by providing contact information for a program director or leader, including institution name, address, contact person, email address, and phone number, to be listed on the ISPE website. Two weeks from the initial email invitation, a follow‐up email invitation was sent to program contacts that had not yet responded. A final reminder was sent in the month after the second contact.

Program contact information that was found to be incorrect (e.g., bounced email addresses, unintended recipient) was noted in a contact log. Of 56 programs listed in the ISPE Program Directory, 42 programs had valid contact information. In cases where the unintended recipient redirected the invitation to the current program representative, the updated contact information was noted and provided to ISPE's Academic Council, and these were counted as successfully contacted programs as contact information was updated. This strategy yielded responses from most programs listed on the ISPE directory with valid contact information, as 41 of 42 programs listed in the directory with valid contact information returned responses. Snowball contacting strategies were then deployed to improve outreach to programs and institutions that were not listed in the directory or had not yet responded. Snowball contacting strategies included direct emails to ISPE Regional Interest Group (RIG) leaders, who were requested to forward the survey invitation and link to all RIG members. Then, Academic Council members were contacted directly via email as well as through the ISPE Exchange website with a request to circulate the survey invitation to known programs. Finally, ISPE members were approached via announcements (in‐person during ISPE meeting symposia as well as posted on ISPE's website) for requests to circulate the survey invitation at the annual 2024 ISPE meeting. Data collection began in August 2023 with the email invitations to all programs listed in the ISPE Program Directory. Snowball sampling strategies were deployed for further data collection between January 2024 and January 2025, where saturation was determined as achieved by the ISPE Academic Council and workgroup members in December 2024. Following review of received responses in an Academic Council meeting in December 2024, it was noted that further responses were no longer being collected from repeated inquiries and this lack of new responses following the most recent round of invitations was the justification for achieving saturation. As a result, the survey was officially closed in January 2025, following one final emailed reminder to RIG leaders and Academic Council members who had not yet confirmed distribution to their networks. The list of pharmacoepidemiology training program participants is available as Supporting Information: Appendix 3.

### 2.4 Analysis

Responses were examined for duplicate participation by programs. There were cases where a program representative responded once from the initial direct, personal invitation delivered via email in 2023 and then the same program representative responded a second time when contacted via snowball sampling strategies in 2024, in which case only one response from that program was included in the count of respondents and in the analysis. Frequencies for all question item response options were calculated. The denominator when calculating response frequency for individual items was defined as the total number of responses received for that question item. When no response was provided to a question item, that item was considered to have missing data. Responses of “Not Applicable” to individual question items were not treated as missing data and, thus, “not applicable” responses were included within item denominators.

Geographical region location of programs was determined by participant response to the question regarding program country location, or in cases when the program country was not provided, then the respondent RIG membership status was used to assign the geographic region location. Bar charts were generated for question items endorsing whether individual competencies were covered in the respondent's training program and were organized into separate plots by competency ‘themes’, which mirror the organization and presentation of individual competencies in the updated core competencies paper. Recommendations on educational content development to address curriculum gaps noted by respondents were compiled from assessment results and presented, as written, by program representative respondents. Thematic analysis by coding of free‐text responses regarding educational needs to support curriculum was initially conducted by one reviewer, who then presented a draft of coded categorical themes to coauthors, where all individual responses were reviewed as aligning or not within the proposed categorical themes by the larger group. Coauthors recommended revisions to the draft coding schema to broaden themes (e.g., combining responses about themes of “writing” and “teaching” to a single theme of “Communication”) and consensus was achieved following initial review.

## 3 Results

**FIGURE 1:**
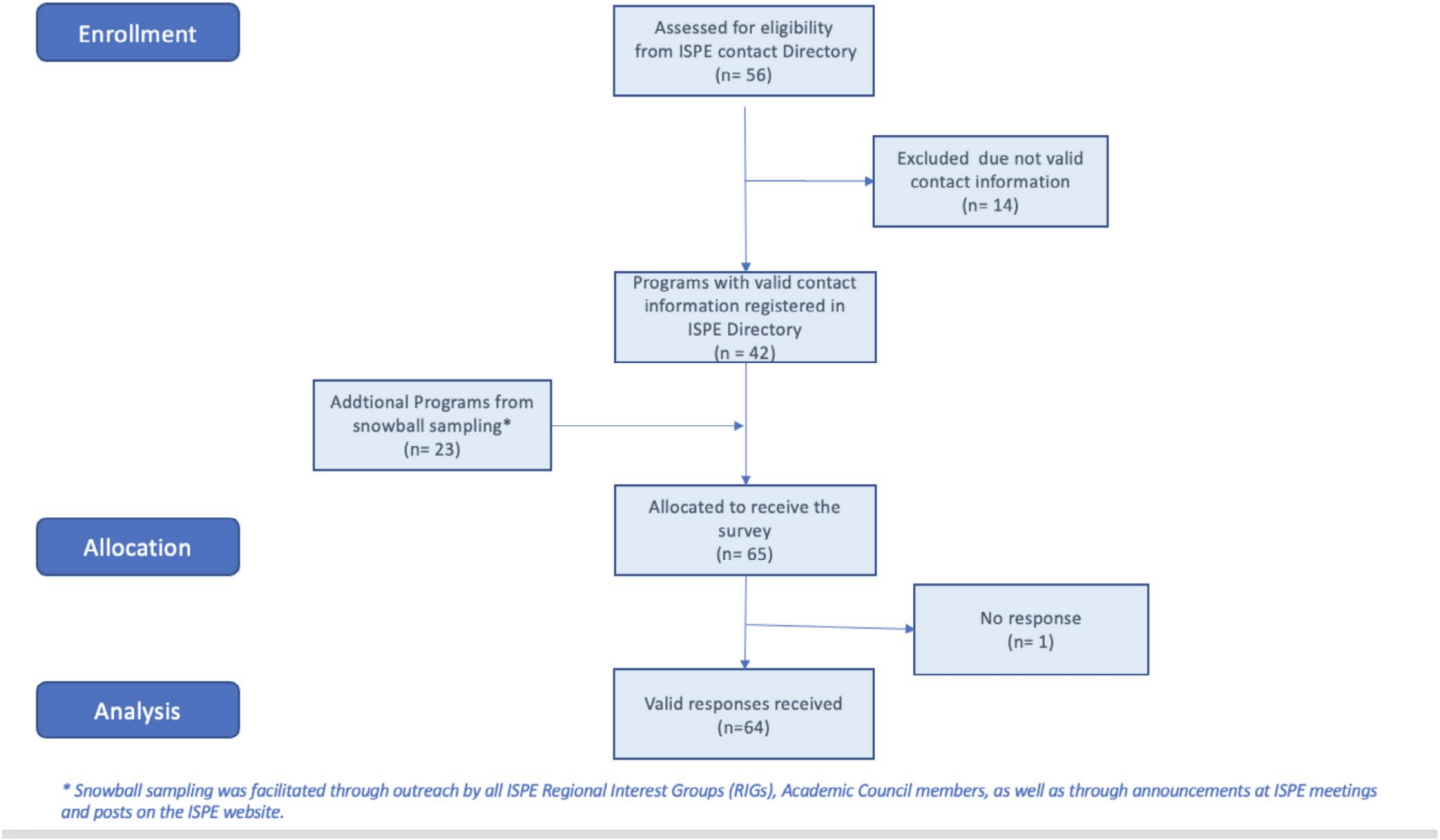
Flow diagram of recruitment strategies and pharmacoepidemiology educational program participation.

Following invitations to all 56 programs listed in the ISPE Program Directory, 41 responses were received from the 42 programs with valid contact information (i.e., 41 of 42, meaning a 97.6% response rate, from the directory entries with valid contact information), and an additional 23 responses were collected afterwards from snowball contact strategies. A total of 64 educational and training programs thus participated by returning a survey response (see Figure 1).

By sector, most programs participating in the survey represented universities or academic institutions (47 of 64, or 73.4%), though two such programs described themselves as collaborations between academic institutions and industry (e.g., “hybrid”) but were primarily considered academic as per their organizational structure. There were 8 programs representing industry (pharmaceutical industry or contract research organizations) and 4 programs from consulting services/agencies and/or health‐systems, comprising a total of 12 industry programs (12 of 64, or 18.8%). The remaining program participants represented government or regulatory authorities (5 of 64, or 7.8%). The majority of program respondents were located in Europe (23 of 64, or 35.9%; see Figures in Supporting Information: Appendix 4), followed by North America (19 of 64, or 29.7%), Latin America (15 of 64, or 23.4%), Asia (5 of 64, or 7.8%), Africa (1 of 64, or 1.6%), and the Gulf Region (1 of 64, or 1.6%).

### 3.1 Curriculum Assessment Results

**FIGURE 2:**
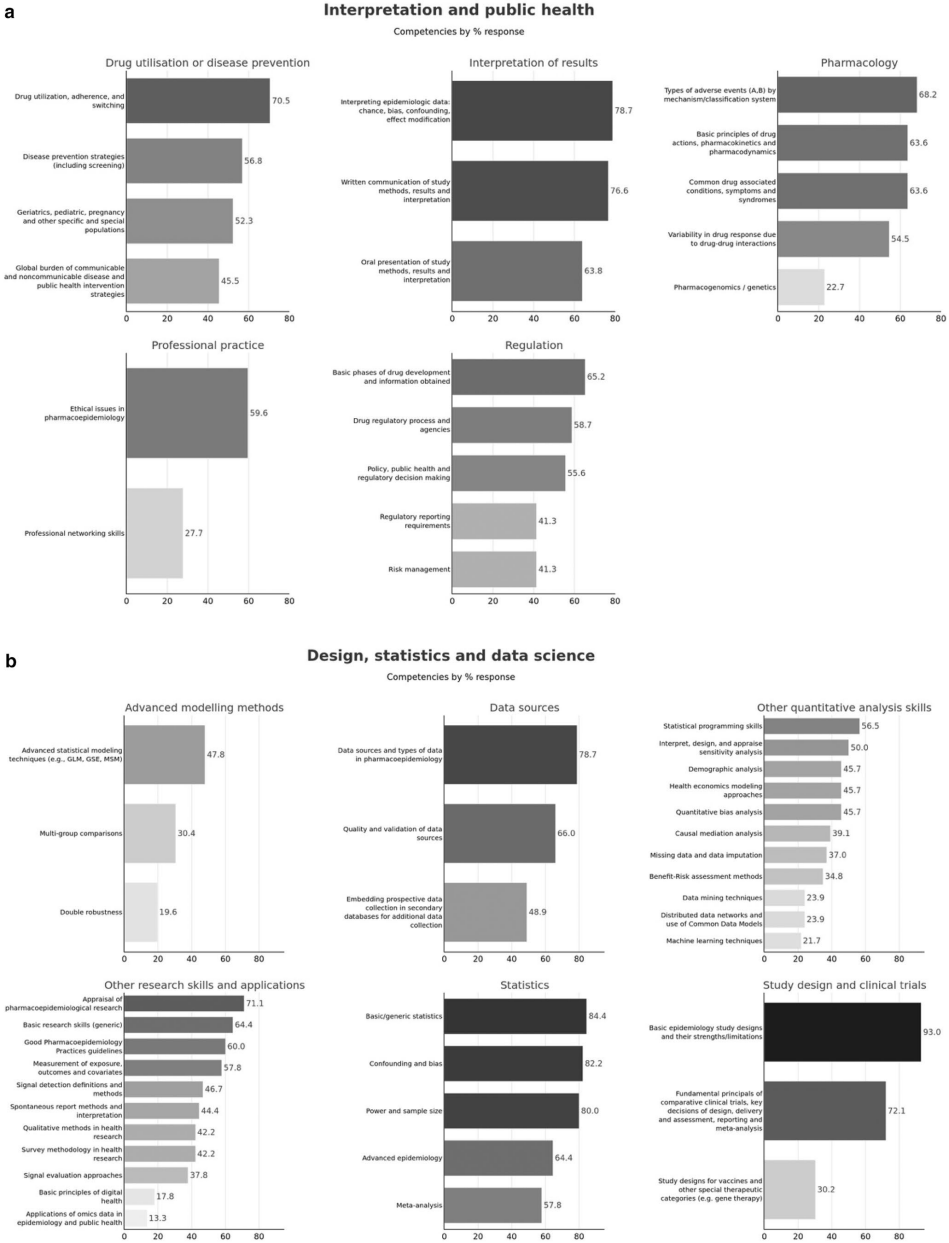
Core competency coverage in all responding pharmacoepidemiology education or training programs (*n* = 64). (a) Interpretation and public health. (b) Design, statistics and data science. *Note:* The series of histograms in (a) and (b) are grouped into the “themes” as presented in the updated core competencies manuscript. Lighter hues of bars represent a lower proportion of program respondents covering that individual competency within their curriculum, while darker hues represent a higher proportion of program respondents covering that individual competency.

Figure 2 provides the overall summary of responses from all programs regarding whether individual core competencies are covered within the respondent program's curriculum. Program participant endorsement of individual competency coverage within their curriculum is represented in the series of grouped histograms using a color gradient, where darker hues represent a higher proportion of respondents covering that individual competency.

**FIGURE 3:**
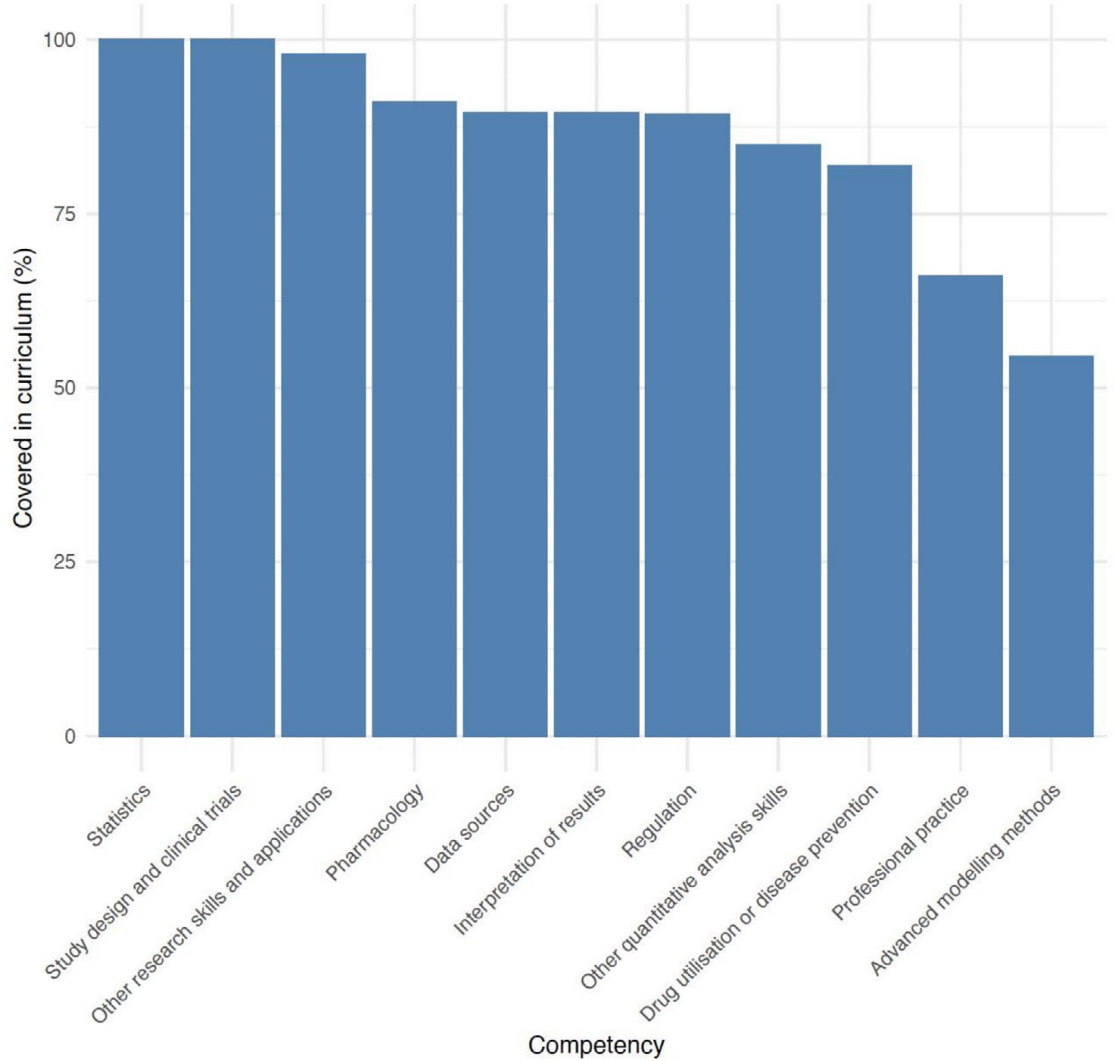
Core competency coverage in all responding pharmacoepidemiology education or training programs (*n* = 64), by competency “Themes.”

The individual core competency covered in curricula by the most program respondents was “Basic epidemiology study designs and their strengths and limitations,” with all program respondents endorsing this competency. The individual core competency covered by the fewest programs (*n* = 1) was “Applications of—omics data in epidemiology and public health.” When organizing individual core competencies within the themes as presented in the updated core competencies manuscript (see Figure 3), the most represented competencies covered in pharmacoepidemiology curricula were competencies within the “Statistics” theme (100%, 45 out of 45 program respondents), and then “Study Design” (100%, 43 out of 43 program respondents). The least represented competencies in themes were within “Advanced Modeling” (54%, 25 out of 46 program respondents), and “Professional Practice” (66%, 31 out of 47 program respondents). In Supporting Information, all individual competencies within each of the 11 themes are plotted via separate histograms for a more granular visual representation of responses (see Supporting Information: Appendix 5). We did not observe notable curriculum gaps between geographic regions.

### 3.2 Educational Needs Assessment Results

Key needs in pharmacoepidemiology curriculum as identified by program representatives centered around four themes: 33.3% (8/24) “Methods (generally),” 33.3% (8/24) “Analytical Skills,” 16.7% (4/24) “Communication (writing, teaching),” and 25.0% (6/24) “Other (applications, new therapies).” Responses with the theme of ‘Methods’ were distinct from responses within the theme of “Analytical skills,” as those responses in the “Methods” theme described competencies related to study conceptualization and design, whereas responses within the theme of “Analytical skills” described competencies related to the applications and practice of specific statistical or analytical tools and programming skills. When program representatives were asked which areas to prioritize in developing standards in pharmacoepidemiology training curriculum, two areas gained the majority of endorsements: “Epidemiology,” with 45.5% of respondents endorsing (20 of 44), and 36.4% of respondents (16 of 44) endorsing “Statistics, Analysis, and Data Science.” Few other areas were endorsed by program respondents.

Program representatives were asked about types of educational content that would best support curriculum development. Content type preference, as ranked by 42 respondents, was: “Learning objectives with pre‐packaged activities” 45.2% (19/42), “Recorded webinars/lectures” 28.6% (12/42), “Example syllabi” 21.4% (9/42), and “Reading lists” 4.8% (2/42). Given these responses, it is recommended that ISPE‐endorsed educational materials consider the content delivery mechanism preferences expressed by program representatives in this needs assessment survey.

Nearly all respondents answered question items that described their program structure, location, and the majority of respondents answered question items describing core competency representation within their curriculum. However, less than half of respondents provided feedback on key educational needs to support development of pharmacoepidemiology curriculum, and among these respondents, all but one were program directors in academic institutions. Additionally, the majority of academic program director respondents reported experience levels in pharmacoepidemiology of > 10 years. No other clear patterns were identified in missingness of item responses by geographic distribution or by sector.

## 4 Discussion

This manuscript serves as a *State of the Field* report for pharmacoepidemiology training, which is needed following the revisions of the definition of pharmacoepidemiology and the updated core competencies. As the 2024–2029 ISPE strategic plan calls for the organization to “build expertise and promote the advancement of pharmacoepidemiological science” (ISPE policy manual, section 1.2) [6], this assessment of curriculum can inform development of educational initiatives that build capacity for pharmacoepidemiology training and facilitate career progression for ISPE members. This capacity building comes at an important time as ISPE's strategic goals include expanding organizational presence and allocating training resources in jurisdictions as well as fields with less traditional pharmacoepidemiology representation.

A major curriculum gap noted by program respondents included lack of training for competencies related to “Advanced modeling.” Individual competencies within “Advanced modeling,” include “Advanced statistical modeling techniques (e.g., Generalized Linear Models [GLM], Generalized Estimating Equations [GSE], Marginal Structural Models [MSM])” as well as “Multi‐group comparisons.” It is noted that most program respondents identifying these gaps in their curriculum also endorsed the need for “learning activities with packaged activities,” suggesting that pragmatic resources, such as access to data and facilitating access to analytical tools or software packages, could be of benefit in addressing this curriculum gap.

Another important curriculum gap noted by program respondents was those individual competencies related to “Professional practice,” which include “Ethical issues in pharmacoepidemiology” and “Professional networking skills.” Though it is unclear based on this assessment what underlying reasons may be driving the curriculum gap for professional practice‐related competencies, what is clear is a notable opportunity for ISPE to leverage already successful initiatives in filling these educational gaps. ISPE's successful mentoring program for new members as well as the ample opportunities provided to ISPE members for events such as free webinars and networking‐focused meetings can serve as natural bridges into ISPE's strategic goals that span member enrichment and pharmacoepidemiology education.

There are several ongoing as well as planned educational and training initiatives that are already available to ISPE members. Notably, the ISPE short‐courses provided to members and/or meeting attendees before the annual meeting include an annually refreshed mix of both advanced methods topics as well as recurring fundamental or foundational pharmacoepidemiology content. ISPE's Education Center, available from pharmacoepi.org, contains a host of regularly updated educational and training resources, including webinars (ISPE‐supported or endorsed) and archived meeting recordings. The official journal for ISPE, *Pharmacoepidemiology and Drug Safety*, also plays an important role in disseminating educational materials. The journal offers a series titled “Core Concepts in Pharmacoepidemiology” that reviews important study design and methods concepts, which are all available open access to the public.

Each of the resources described above can provide learners and educators with a pathway to get practical curriculum resources now, but ISPE has further opportunities to participate in training resource development. The Education Committee, responsible for developing and implementing many such initiatives, is open to participation from all ISPE members. Additionally, the 2024–2029 ISPE strategic plan calls for expanding ISPE's pharmacoepidemiology educational and training efforts, and several new initiatives are in development.

We offer the following recommendations to address the gaps identified in pharmacoepidemiology curriculum and educational needs, as informed by this assessment of curriculum alignment with core competencies (see Table 1): (1) Develop and support ISPE‐endorsed educational materials for each of the individual core competencies for which limited programs report including in current pharmacoepidemiology curriculum (specifically, training for methods and analytical techniques such as machine learning applications and advanced modeling techniques, training for science communication, and training in understanding ethical issues); (2) Enhance advertising of current ISPE initiatives to improve member engagement in professional development opportunities (specifically, to hone competencies for professional networking and science communication); and, (3) Consider alternative strategies for ISPE‐endorsed or provided educational and training content delivery to promote ‘enduring’ access to pre‐packaged activities that include materials to supplement current reading lists and webinar‐provided trainings (i.e., learning objectives paired with: demonstrations, sample data, sample programming code, and problem sets with annotated solutions).

**TABLE 1:** Recommendations to ISPE.

Recommendation to ISPE	Need addressed by the recommendation	Alignment with strategic goals
Develop and support ISPE‐endorsed educational materials for core competencies for which there are coverage gaps	Supports are needed in the following areas: Machine learning applications Advanced modeling techniques Science communication Understanding ethical issues	“Prepare” (i.e., Capacity building)
Enhance advertising of current ISPE initiatives to improve member engagement	Increase opportunities to hone competencies for professional networking and science communication	“Prepare” (i.e., Capacity building); “Empower”
Consider alternative strategies for ISPE‐endorsed educational content delivery to promote ‘enduring’ access to more comprehensive pre‐packaged activities	Training content standards should extend beyond required learning objectives, where activities are paired with demonstrations, sample data, sample programming code, and problem sets with annotated solutions	“Prepare” (i.e., Capacity building); “Empower”

Our first recommendation is in alignment with the 2024–2029 ISPE strategic plan's goal to “Prepare”; specifically, this recommendation aligns with goal 6, which is to “… create diverse development opportunities for all stages of career …,” and can further support ISPE's mission of capacity building in pharmacoepidemiology. Our second and third recommendations are in alignment with ISPE's strategic plan goals to “Prepare” as above, and also with the goal to “Empower,” as diverse perspectives and geographic regions would need to be represented in such educational content development efforts in order to tailor communication for regional context and to identify regional curriculum needs as they evolve.

### 4.1 Limitations

Though the development team represents ISPE members who are leaders in key groups, and representation from institutions around the globe (South America, North America, Europe, Africa, Australia), representation in responses from training programs and institutions does not appear to be comprehensive, and it is unclear that program census was achieved. As the ISPE Program Directory is the only official measure of educational and training programs known to the authors, it is unclear how many pharmacoepidemiology programs with ISPE participating members are not represented among the final survey participants; however, we note that all pharmacoepidemiology programs, except one, with correct contact information within the ISPE Program Directory participated in this assessment. The reliance on ISPE's Program Directory as a primary source for program recruitment into this curriculum assessment survey may represent a source of selection bias as programs must proactively opt‐in to the registry and then are responsible for updating and maintaining program contact information with ISPE leadership. Though proactive outreach to ISPE Programs and individual Members beyond those listed in the ISPE Program Directory served to partially mitigate selection bias, it is unclear to what extent those efforts were successful. We also note that the addition of the snowball sampling approach following our use of the ISPE contact directory for survey invitation outreach potentially introduced bias in which pharmacoepidemiology training program leaders who had established networks with ISPE leaders and study contributors may have been more likely to receive the invitation to participate. Thus, the extent to which we comprehensively identified and included pharmacoepidemiology training programs across the globe remains unclear. Representation in responses from programs, however, does somewhat reflect the diverse body of ISPE members, and so it should also be considered that program representative respondents may prioritize educational needs differently by region and sector, which suggests that educational needs should be more regularly evaluated as this project can only provide recommendations for the current landscape of pharmacoepidemiology training and educational needs. Relatedly, it should be noted that representatives from education and training programs may have differing views on educational needs in curriculum. Therefore, surveying other representatives from the same programs might have yielded different results when asked about educational needs and content delivery preferences. It should also be noted that participation in qualitative, free‐text question items asking respondents to describe educational needs was lower, suggesting that respondent burden for participating in this initiative could be reduced by limiting free‐text question items. Future assessment of pharmacoepidemiology curriculum could include surveys of all individual ISPE members rather than through solicited invitation from single program representatives to reach a broader audience and more diverse representation of educational needs as identified by pharmacoepidemiologists across sectors in practice as well as in training.

## 5 Conclusions

This global assessment of pharmacoepidemiology curricula and reported educational needs reveals significant gaps in current training programs, particularly in advanced analytical methods and professional practice. ISPE is uniquely positioned to address these gaps by developing targeted educational resources that enhance both methodological rigor and practical skills. Future efforts should align with the expressed preferences of training programs, which favor structured materials such as learning objectives with pre‐packaged activities over more passive resources like reading lists.

## Funding

This group received funding from ISPE's funded manuscript program following proposal review to make this paper available via open access.

## Disclosure

Preliminary results were presented during a workshop at the 2024 ISPE Annual Meeting. Final results were presented as a poster at the ISPE Annual Meeting in August of 2025.

## Ethics Statement

This research is not considered human subjects research and is exempt from institutional review board review. All participation from program representatives in surveys was voluntary and responses do not represent individual participants. No program representatives that described their curriculum and educational gaps were asked to submit personally identifying information.

## Conflicts of Interest

Xiaojuan Li has received support via NIH K01AG073651. Employment disclosures include: Vicki Osborne is an employee of Jazz Pharmaceuticals with stock and/or options in the company. Ryan Chung is an employee of Lane, Clark and Peacock, LLP. Deborah Layton has received a salary from PEPI Consultancy Limited and has received funds from AstraZeneca, Ariello and Annexon Biosciences. She was also an employee of Lane, Clark & Peacock LLP UK. Filion received personal fees from Regeneron and Statlog, both unrelated to the present work. The other authors declare no conflicts of interest.

## Supporting information


**Appendix 1** Pharmacoepidemiology curriculum assessment and educational needs survey instrument (attached).
**Appendix 2**. Pharmacoepidemiology core competencies endorsed by ISPE and competencies mapped to survey items.
**Appendix 3**. List of participating pharmacoepidemiology training programs.
**Appendix 4**. Global representation of pharmacoepidemiology programs in the curriculum and educational needs assessment (*n* = 64).
**Appendix 5**. Plots representing individual core competency coverage within educational program curriculum as organized into competency themes.

**Data S1:** Supporting Information.

## References

[1] “ISPE Overview,” (2024), https://www.pharmacoepi.org/about‐ispe/overview/.

[2] J. K. Jones , H. H. Tilson , and J. D. Lewis , “Pharmacoepidemiology: Defining the Field and Its Core Content,” Pharmacoepidemiology and Drug Safety 21, no. 7 (2012): 677–689. 22488843 10.1002/pds.3198

[3] V. Osborne , A. Goodin , J. Brown , et al., “Updated Core Competencies in Pharmacoepidemiology to Inform Contemporary Curricula and Training for Academia, Government, and Industry,” Pharmacoepidemiology and Drug Safety 33, no. 4 (2024): e5789. 38629216 10.1002/pds.5789

[4] International Society for Pharmacoepidemiology (ISPE) , “About ISPE,” accessed June 1, 2025, https://www.pharmacoepi.org/about‐ispe/overview/.

[5] S. S. McMilan , M. King , and M. P. Tullet , “How to Use the Nominal Group and Delphi Techniques,” International Journal of Clinical Pharmacy 38, no. 3 (2016): 655–662. 26846316 10.1007/s11096-016-0257-x PMC4909789

[6] International Society for Pharmacoepidemiology (ISPE) , “ISPE Strategic Plan 2024–2029,” accessed June 1, 2025, https://www.pharmacoepi.org/strategic‐initiatives/ispe‐strategic‐plan‐2024‐2029/.

