# Supplementary material for: Curriculum in Pharmacoepidemiology Training Programs: A Cross‐Sectional Study to Assess Educational Needs and Alignment With Core Competencies
Source: Pharmacoepidemiol Drug Saf. 2026 Mar 26;35(4):e70351. doi: 10.1002/pds.70351 (PMC13021568; doi:10.1002/pds.70351)

**SUPPLEMENTAL MATERIALS**

**Appendix 1:** Pharmacoepidemiology Curriculum Assessment and Educational Needs Survey Instrument (Attached)

**Appendix 2:** Pharmacoepidemiology Core Competencies Endorsed by ISPE and Competencies Mapped to Survey Items

| Core Competency* | Endorsed by ISPE | Included in Curriculum Assessment Survey |
| --- | --- | --- |
| Advanced statistical modeling techniques (e.g., GLM, GSE, MSM) | Yes | Yes |
| **Double robustness** | **No** | Yes |
| Multi-group comparisons | Yes | Yes |
| Meta-analysis | Yes | Yes |
| Advanced epidemiology | Yes | Yes |
| Basic/generic statistics | Yes | Yes |
| Confounding and bias | Yes | Yes |
| Power and sample size | Yes | Yes |
| Embedding prospective data collection in secondary databases for additional data collection | Yes | Yes |
| Data sources and types of data in pharmacoepidemiology | Yes | Yes |
| Quality and validation of data sources | Yes | Yes |
| Written communication of study methods, results, and interpretation | Yes | Yes |
| Oral presentation of study methods, results, and interpretation | Yes | Yes |
| Interpreting epidemiologic data: chance, bias, confounding, effect modification | Yes | Yes |
| Common drug associated conditions, symptoms, and syndromes | Yes | Yes |
| Types of adverse events (A,B) by mechanism/classification system | Yes | Yes |
| Basic principles of drug actions, pharmacokinetics, and pharmacodynamics | Yes | Yes |
| Variability in drug response due to drug-drug interactions | Yes | Yes |
| Pharmacogenomics/genetics | Yes | Yes |
| Regulatory reporting requirements | Yes | Yes |
| Basic phases of drug development and information obtained | Yes | Yes |
| Drug regulatory process and agencies | Yes | Yes |
| Risk management | Yes | Yes |
| Basic epidemiology study designs and their strengths/limitations | Yes | Yes |
| Study designs for vaccines and other special therapeutic categories (e.g., gene therapy) | Yes | Yes |
| Fundamental principals of comparative clinical trials, key decisions of design, delivery and assessment, reporting and meta-analysis | Yes | Yes |
| Benefit-Risk assessment methods | Yes | Yes |
| Causal mediation analysis | Yes | Yes |
| Distributed data networks and use of Common Data Models | Yes | Yes |
| Data mining techniques | Yes | Yes |
| Demographic analysis | Yes | Yes |
| Health economics modeling approaches | Yes | Yes |
| Machine learning techniques | Yes | Yes |
| Missing data and imputation | Yes | Yes |
| Statistical programming skills | Yes | Yes |
| Quantitative bias analysis | Yes | Yes |
| Interpret, design, and appraise sensitivity analysis | Yes | Yes |
| Appraisal of pharmacoepidemiological research | Yes | Yes |
| Policy, public health, and regulatory decision making | Yes | Yes |
| Good pharmacoepidemiology practices guidelines | Yes | Yes |
| Applications of omics data in epidemiology and public health | Yes | Yes |
| Qualitative methods in health research | Yes | Yes |
| Basic research skills (generic) | Yes | Yes |
| Spontaneous report methods and interpretation | Yes | Yes |
| Signal detection definitions and methods | Yes | Yes |
| Signal evaluation approaches | Yes | Yes |
| Basic principles of digital health | Yes | Yes |
| Survey methodology in health research | Yes | Yes |
| Measurement of exposure, outcomes and covariates | Yes | Yes |
| Global burden of communicable and noncommunicable disease and public health intervention strategies | Yes | Yes |
| Disease prevention strategies (including screening) | Yes | Yes |
| Drug utilization, adherence, and switching | Yes | Yes |
| Geriatrics, pediatric, pregnancy and other specific and special populations | Yes | Yes |
| Ethical issues in pharmacoepidemiology | Yes | Yes |
| Professional networking skills | Yes | Yes |
| **Evaluation of effectiveness and impact** | Yes | **No** |
| **Understanding of biological mechanisms** | Yes | **No** |

**Note:* The list of final core competencies endorsed by ISPE underwent slight revisions prior to the release of this curriculum needs assessment survey. Hence, we map the final list of individual core competencies as published in Osborne, et al. (2024) to all individual competencies assessed in the educational needs survey. Discrepant entries are bolded.

**Appendix 3:** List of Participating Pharmacoepidemiology Training Programs

| Program or Institution Name | Country | Sector |
| --- | --- | --- |
| Aarhus University | Denmark | Academic |
| Agencia Nacional de Vigilencia Saniteria (Anvisa) | Brazil | Government |
| AllStripes Research | United States of America | Industry |
| Amgen (France) | France | Industry |
| Amgen (United Kingdom) | United Kingdom of Great Britain and Northern Ireland | Industry |
| AstraZeneca | France | Industry |
| Boehringer Inhelheim | Germany | Industry |
| Boston University School of Public Health | United States of America | Academic |
| Brasilia University | Brazil | Academic |
| Cognizant | Sweden | Industry |
| Consulting (private) | Colombia | Consulting |
| Drug Safety Research Unit | United Kingdom of Great Britain and Northern Ireland | Academic |
| Federal University of Bahia | Brazil | Academic |
| Federal University of Ceare | Brazil | Academic |
| Federal University of Sao Paulo | Brazil | Academic |
| Gilead sciences | Turkey | Industry |
| Harvard TH Chan School of Public Health | United States of America | Academic |
| Health Canada | Canada | Government |
| Hoffmann-La Roche Ltd | Switzerland | Industry |
| Inserm | France | Academic |
| Instituto de Salud Publica de Chile | Chile | Government |
| Instituto Mexicano Del Seguro Social | Mexico | Government |
| Instituto Nacional de Salud | Peru | Government |
|  |  |  |
| IQVIA (Netherlands) | Netherlands | Consulting |
| IQVIA (United States) | United States of America | Consulting |
| Johns Hopkins University | United States of America | Academic |
| JSS College of Pharmacy, JSS Academy of Higher Education and Research | India | Academic |
| Keio University | Japan | Academic |
| Lane Clark & Peacock (LCP) | United Kingdom of Great Britain and Northern Ireland | Consulting |
| London School of Hygiene & Tropical Medicine | United Kingdom of Great Britain and Northern Ireland | Academic |
| Mafra Distribuidora | Brazil | Hybrid |
| McGill University | Canada | Academic |
| McMaster University | Canada | Academic |
| National Cheng Kung University | Taiwan | Academic |
| Nelson Mandela University | South Africa | Academic |
| Novartis and the University of Basel | Switzerland | Hybrid (based in Academic) |
| Oswaldo Cruz Foundation (Fiocrux | Brazil | Academic |
| Padjadjaran University | Indonesia | Academic |
| Rio de Janeiro Federal University | Brazil | Academic |
| UiT The Arctic University of Norway | Norway | Academic |
| Universidad Nacional de Colombia | Colombia | Academic |
| Universidade Federal de Minas Gerais | Brazil | Academic |
| Universite Laval (Faculty of pharmacy) | Canada | Academic |
| University of Bordeaux | France | Academic |
| University of Copenhagen | Denmark | Academic |
| University of Florida | United States of America | Academic |
| University of Illinois Chicago | United States of America | Academic |
| University of Kentucky | United States of America | Academic |
| University of Ljubljana, Faculty of Pharmacy | Slovenia | Academic |
| University of Manchester | United Kingdom of Great Britain and Northern Ireland | Academic |
| University of North Carolina Chapel Hill | United States of America | Academic |
| University of Novi Sad | Serbia | Academic |
| University Paris Saclay | France | Academic |
| University of Pennsylvania | United States of America | Academic |
| University of the Philippines Manila | Philippines | Academic |
| University of Rhode Island | United States of America | Academic |
| University of Sorocaba | Brazil | Academic |
| University of South Carolina | United States of America | Academic |
| University of Szeged | Hungary | Academic |
| University of Texas at Austin | United States of America | Academic |
| University of Utah | United States of America | Academic |
| University of Washington | United States of America | Academic |
| Uppsala University | Sweden | Academic |
| Utrecht University | Netherlands | Academic |

**Appendix 4:** Global Representation of Pharmacoepidemiology Programs in the Curriculum and Educational Needs Assessment (n=64)


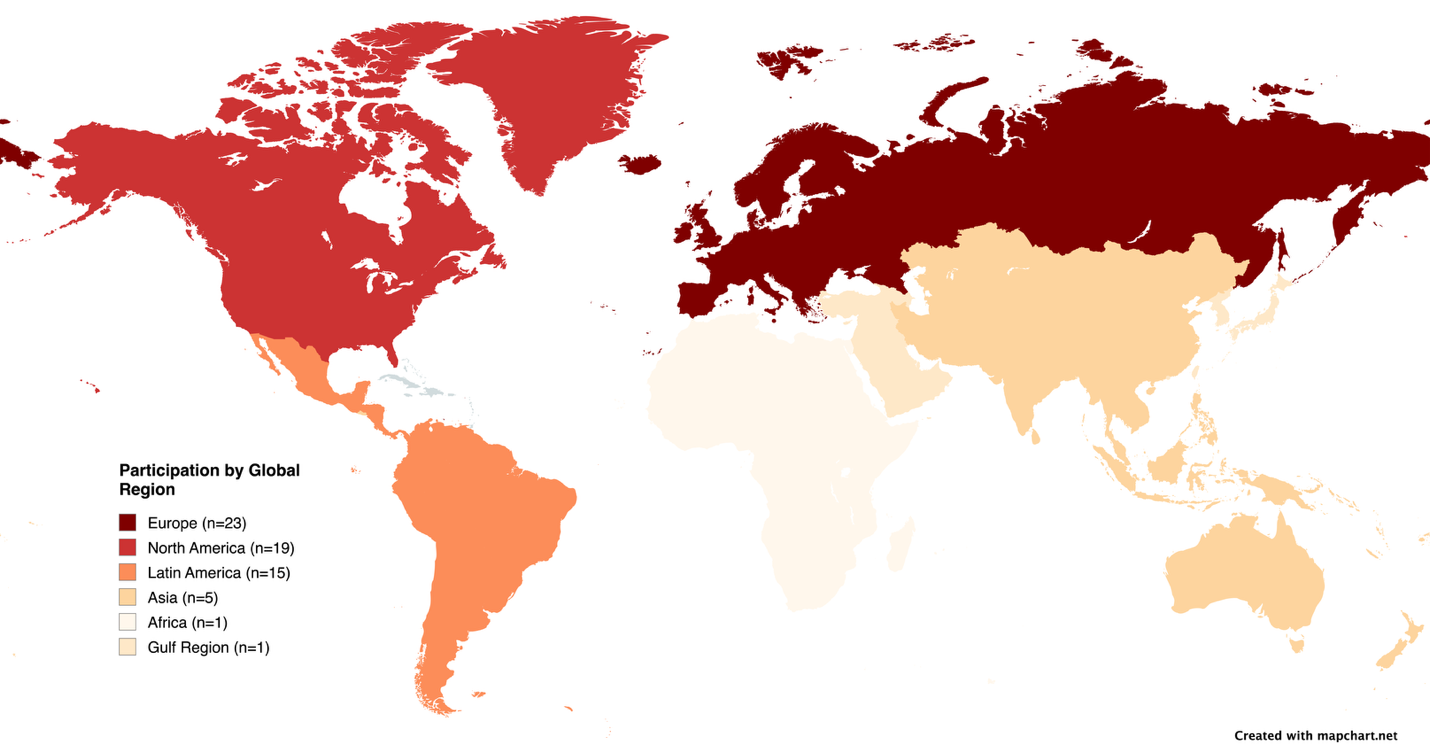


**Appendix 5:** Plots Representing Individual Core Competency Coverage within Educational Program Curriculum as Organized into Competency Themes


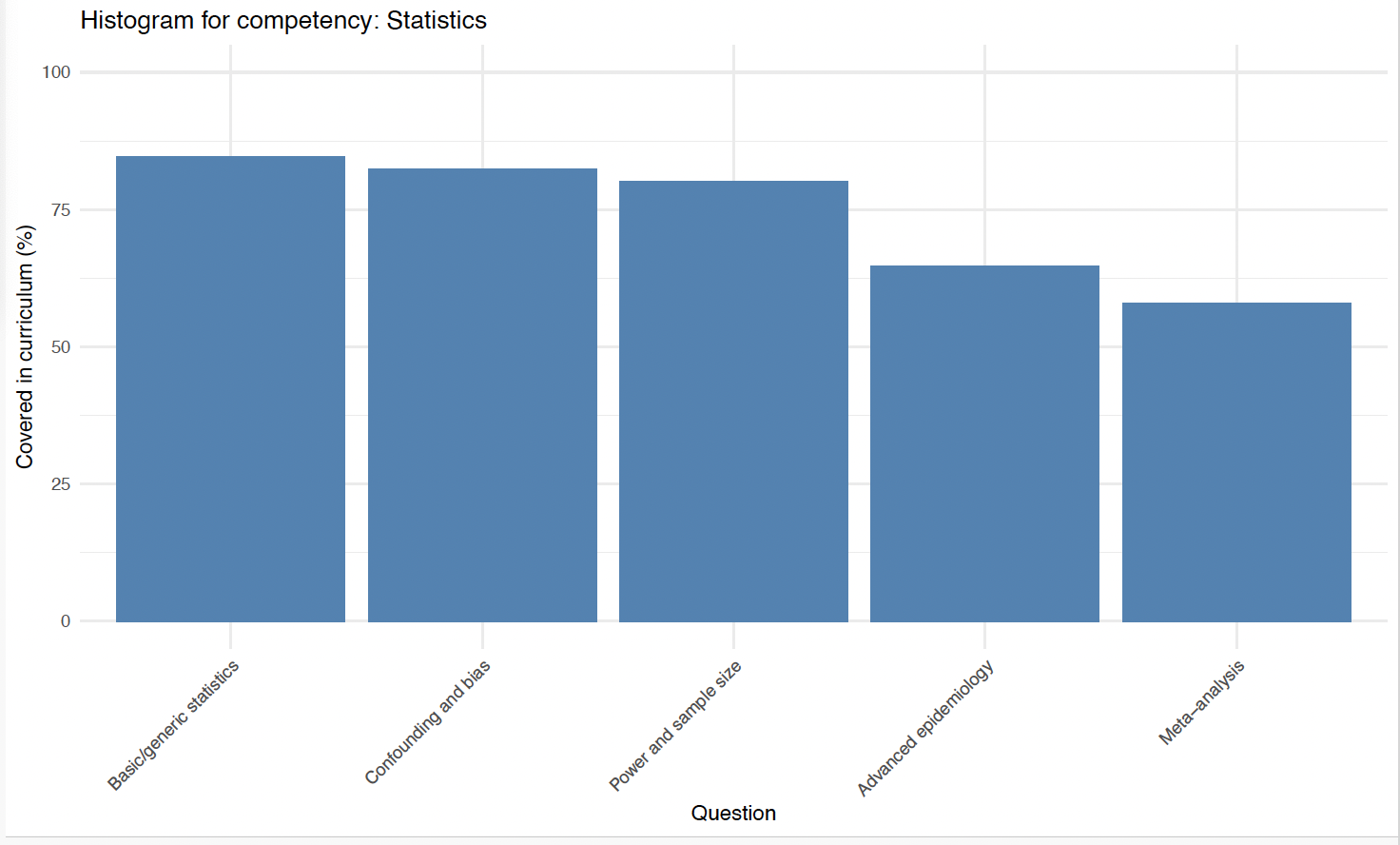


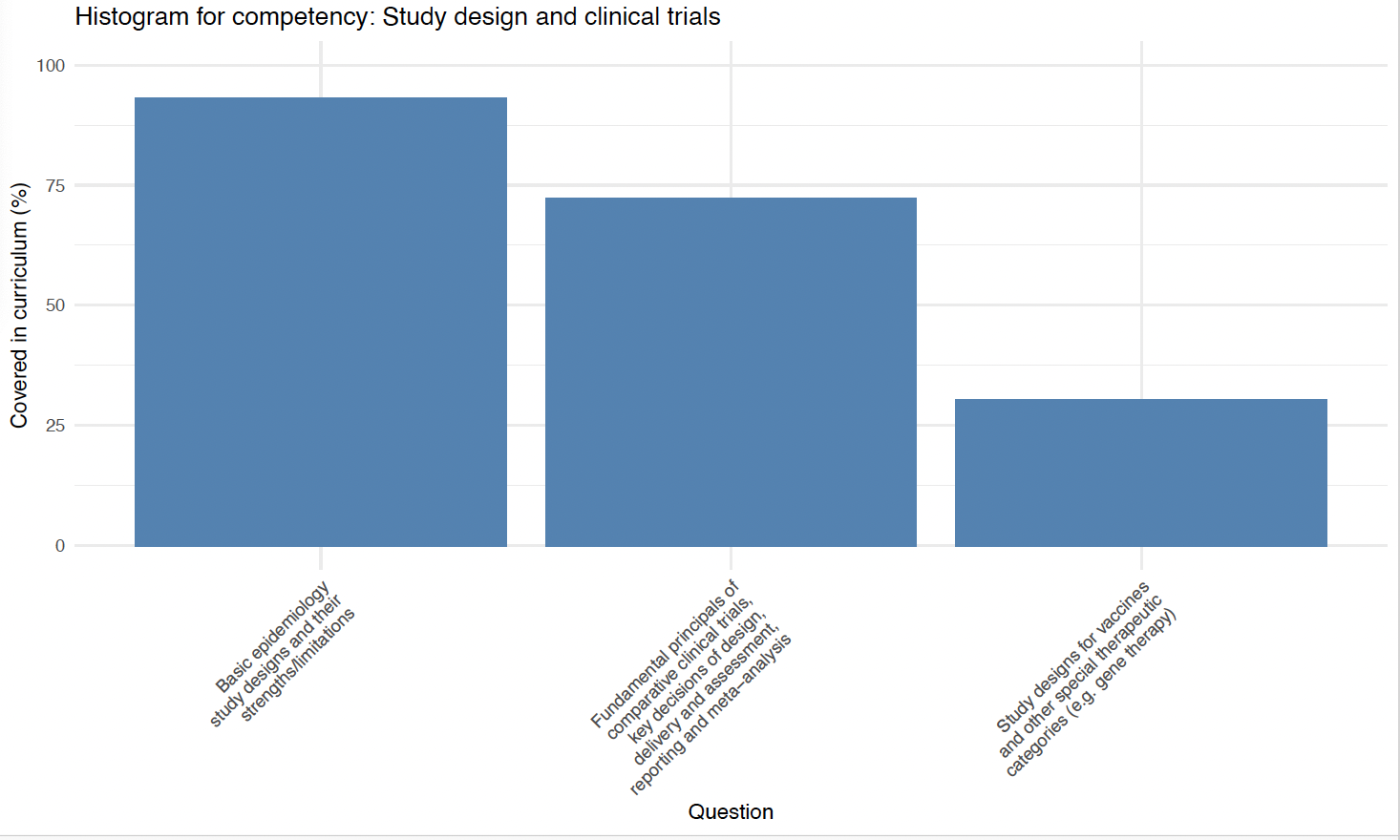


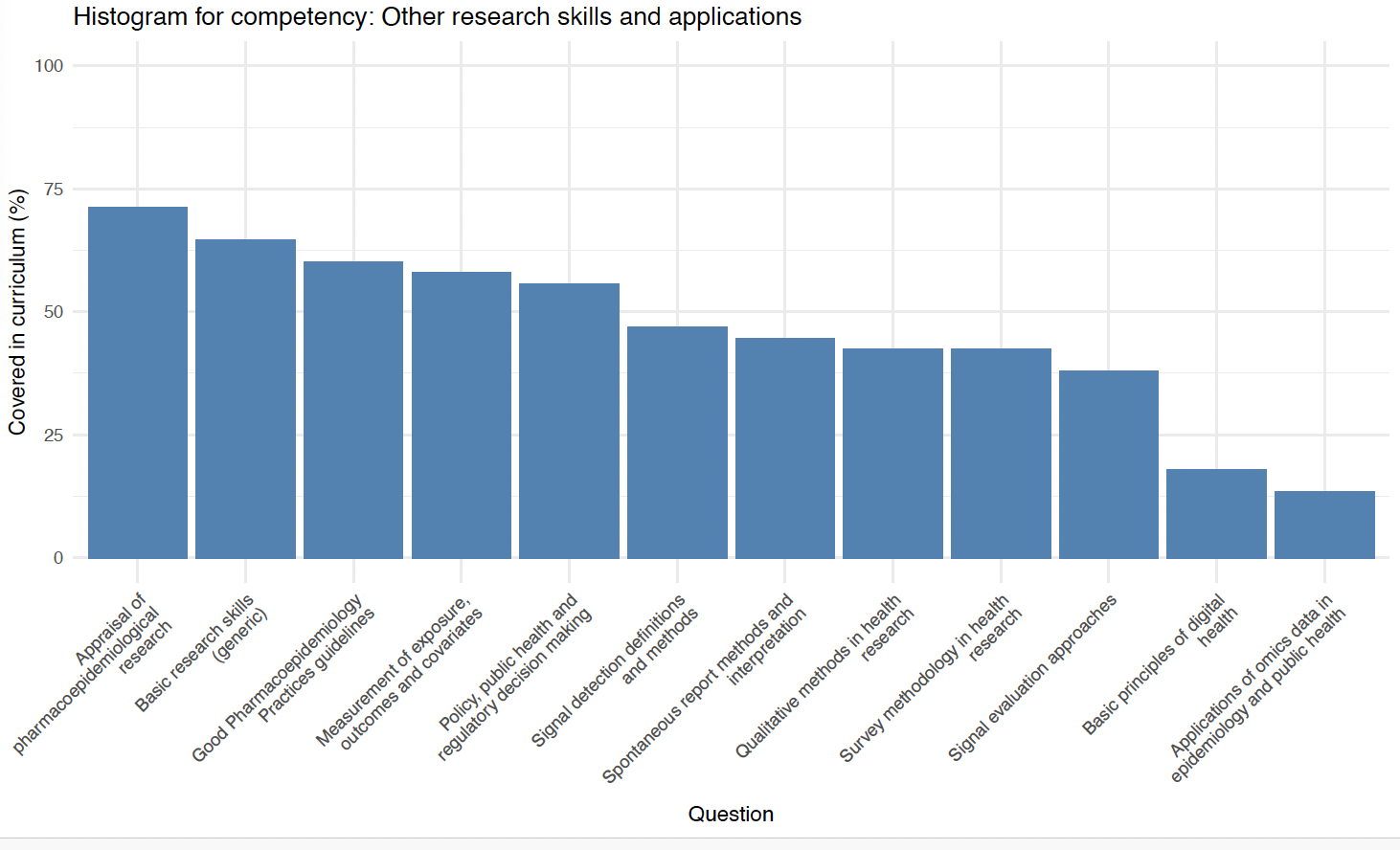


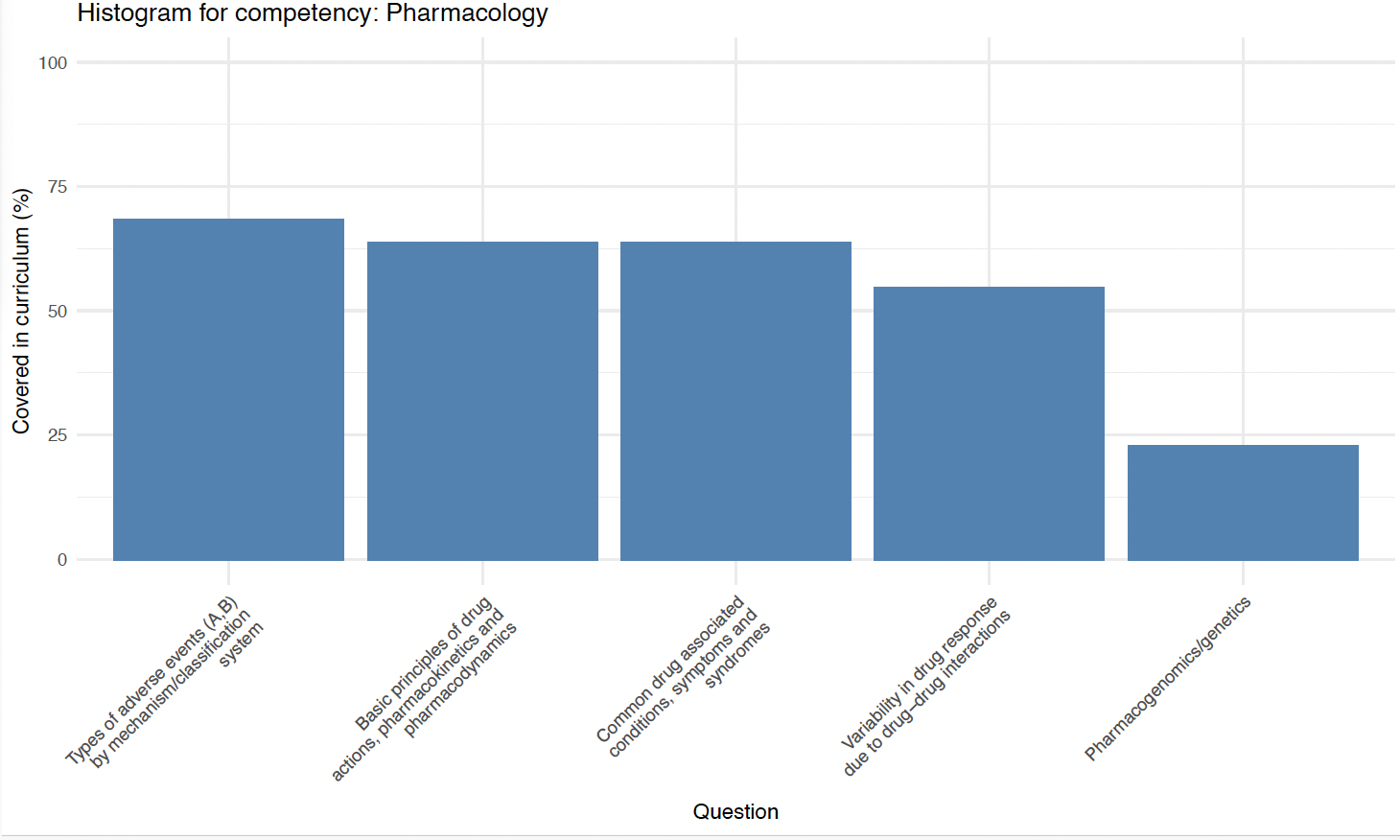


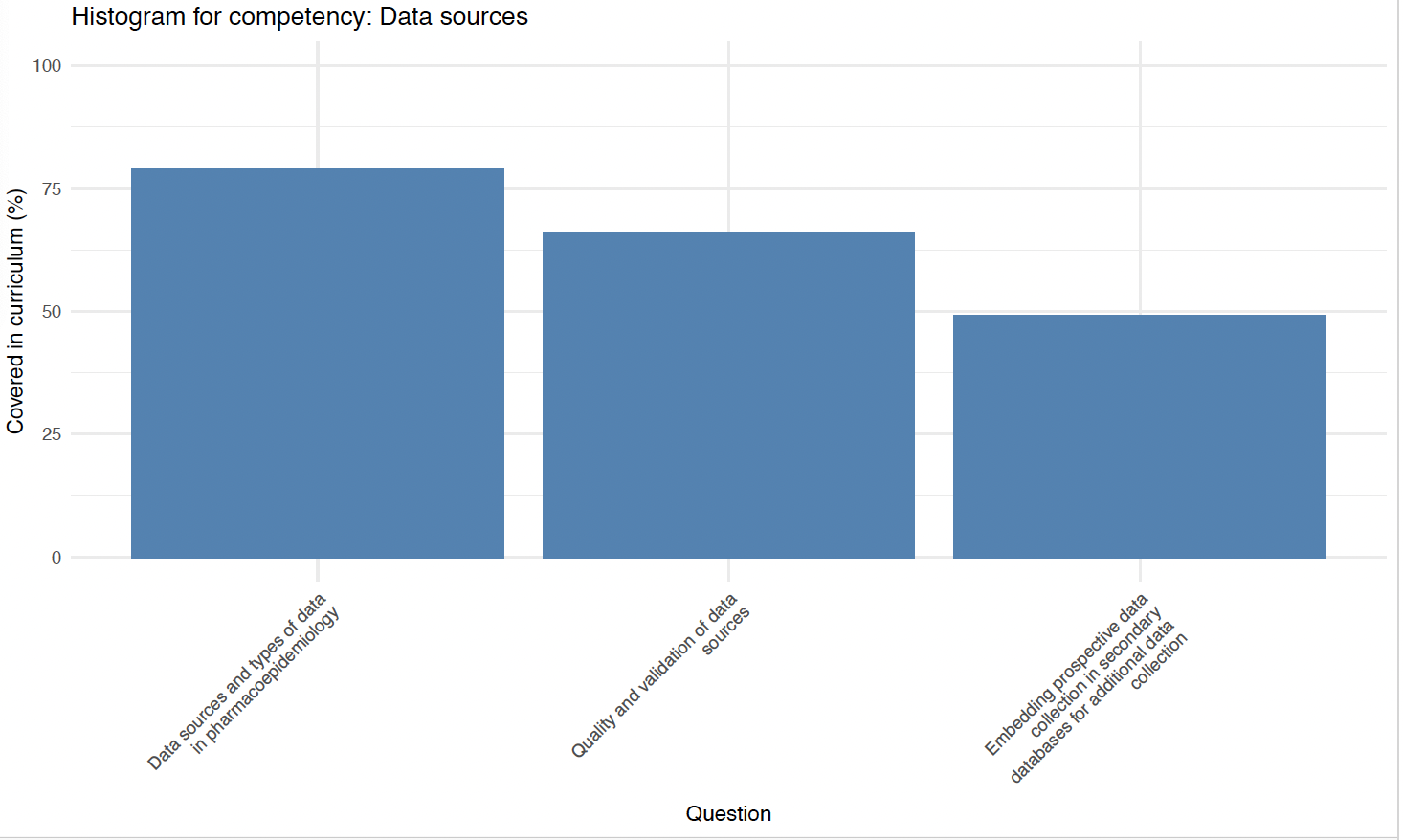


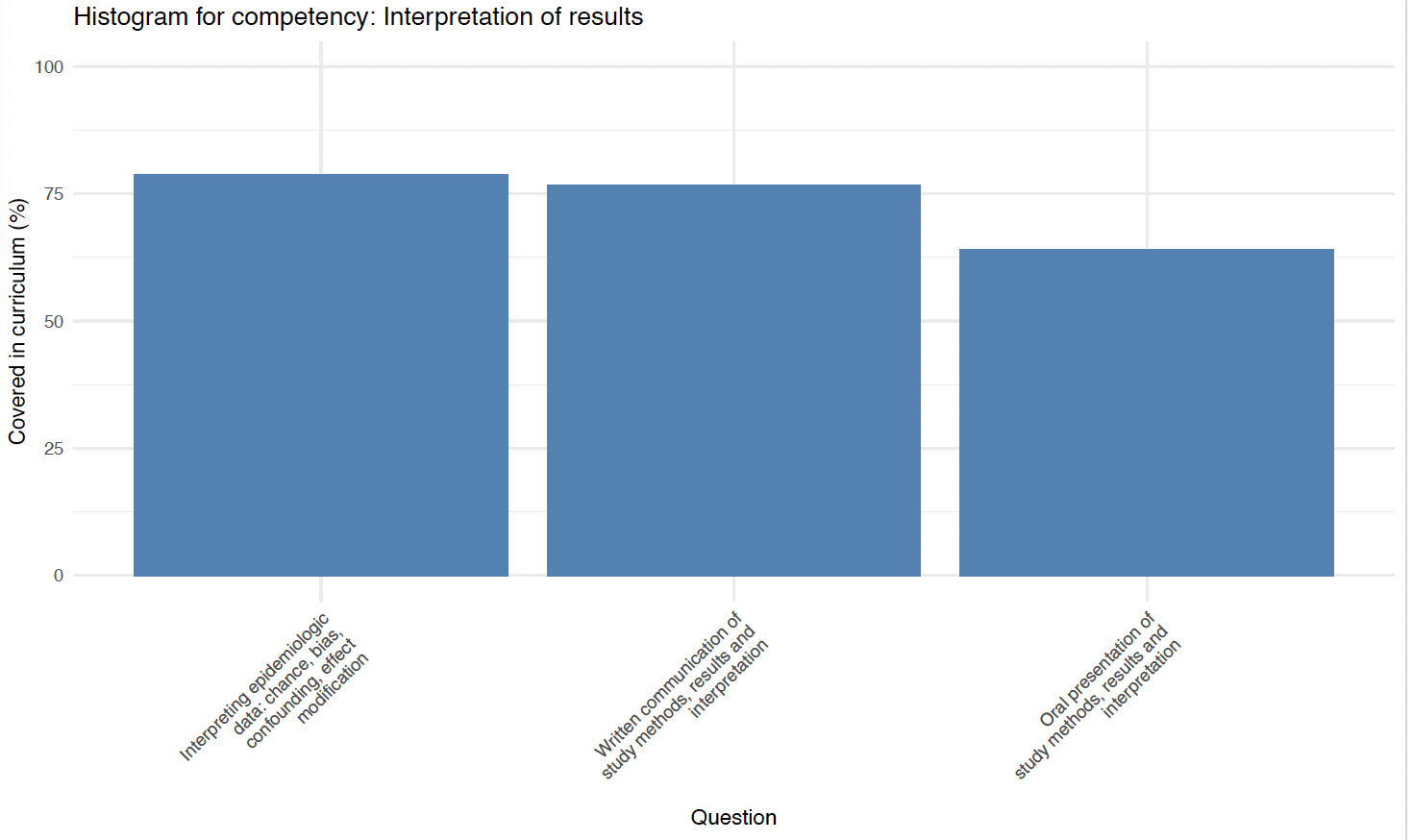


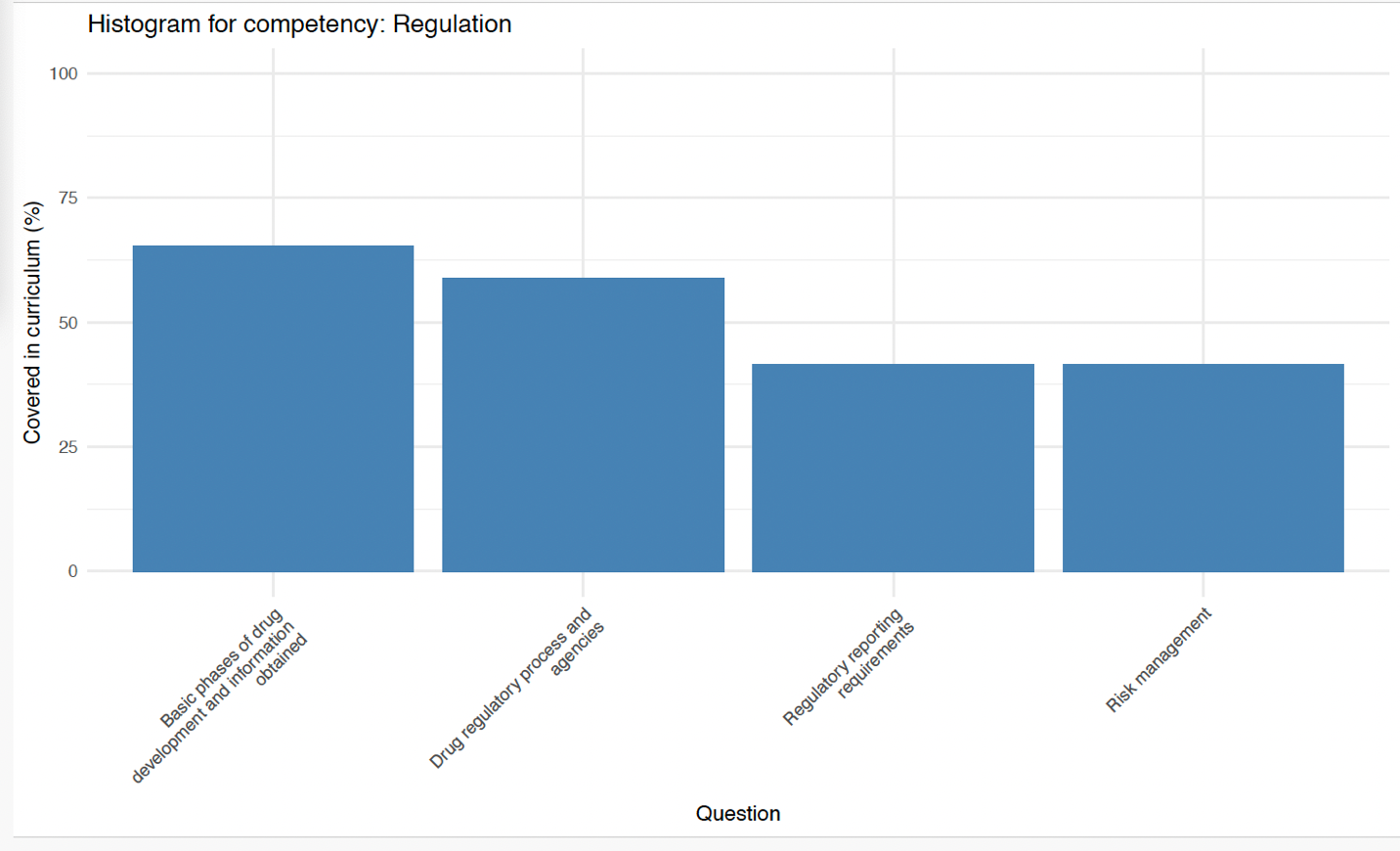


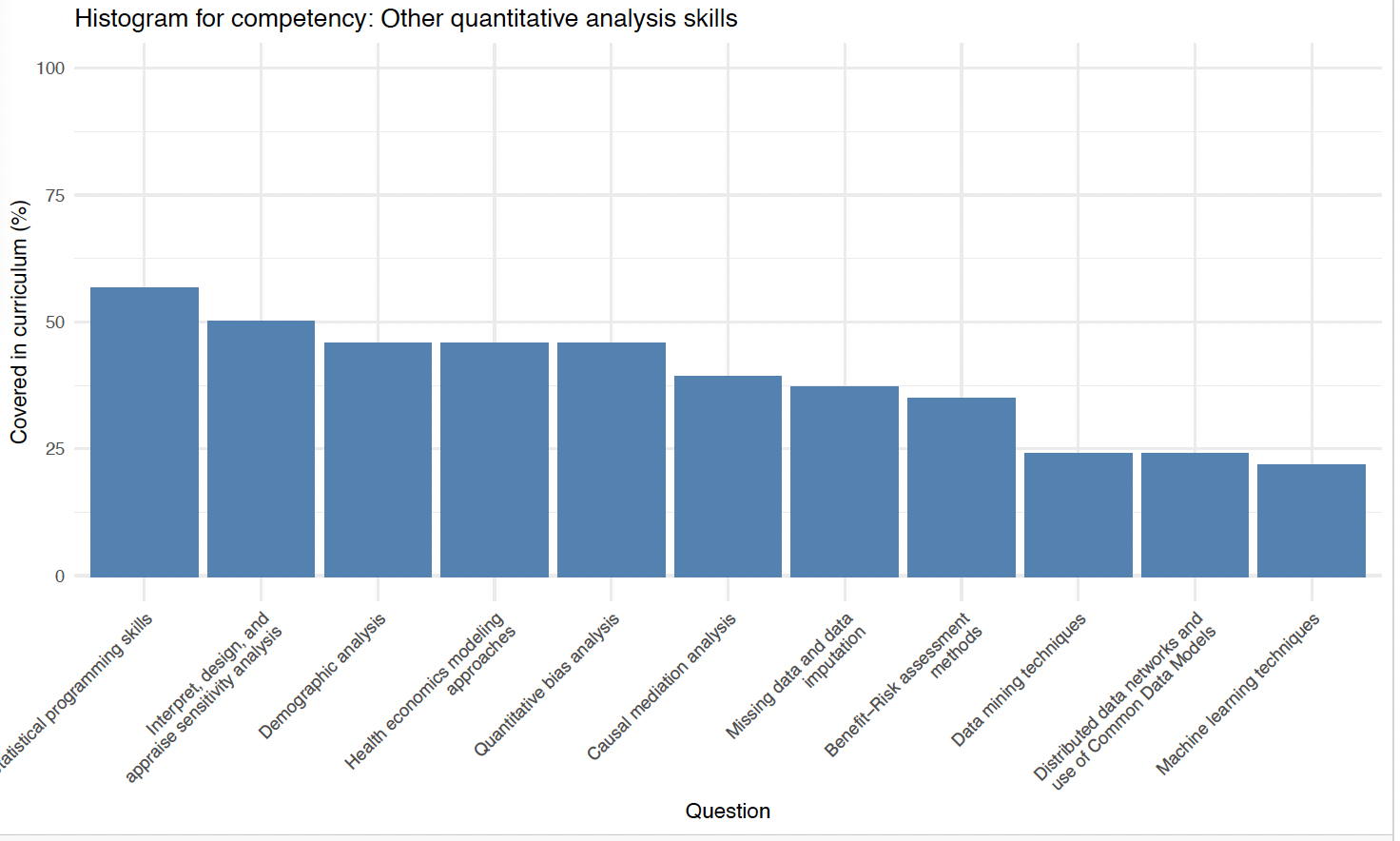


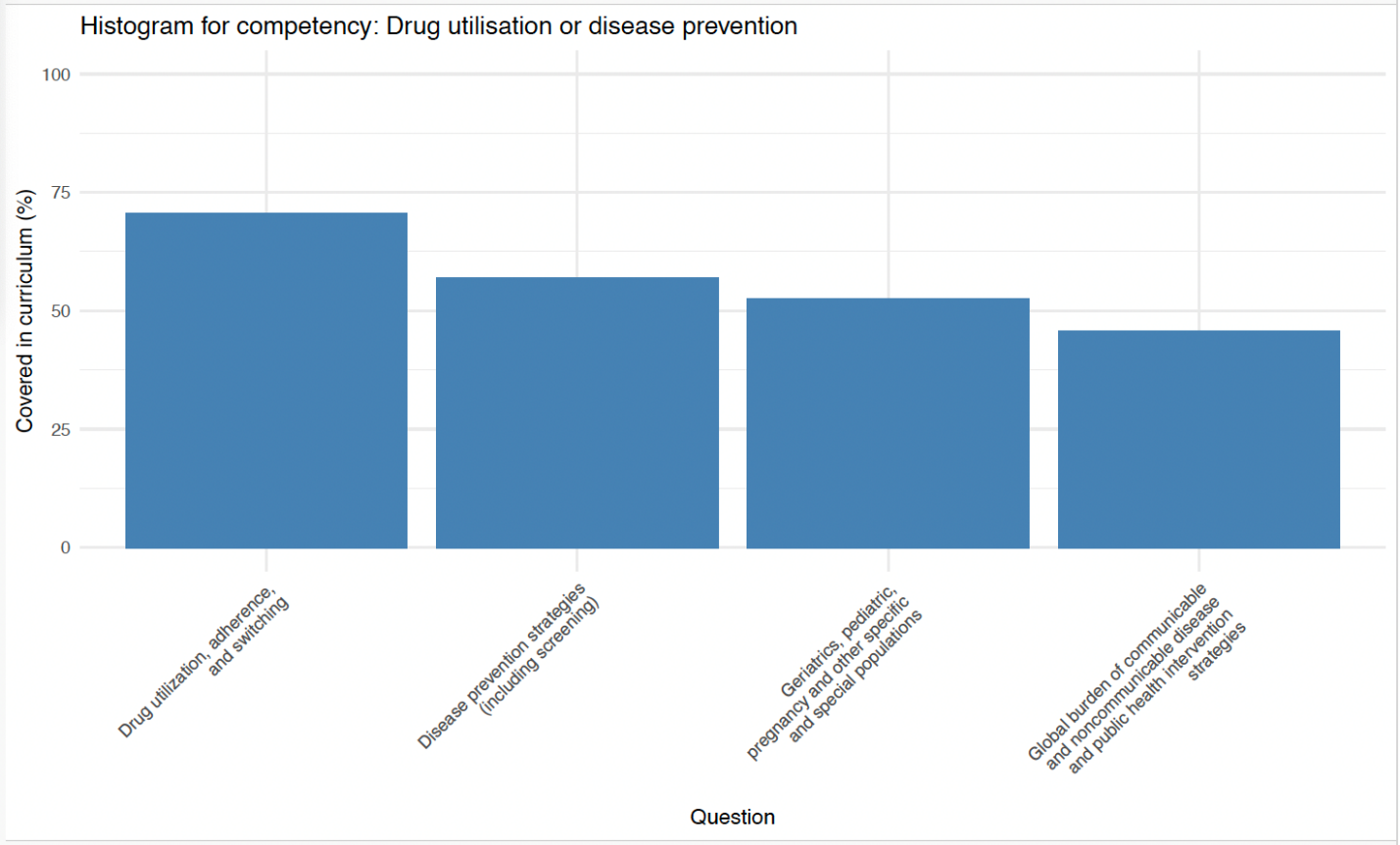


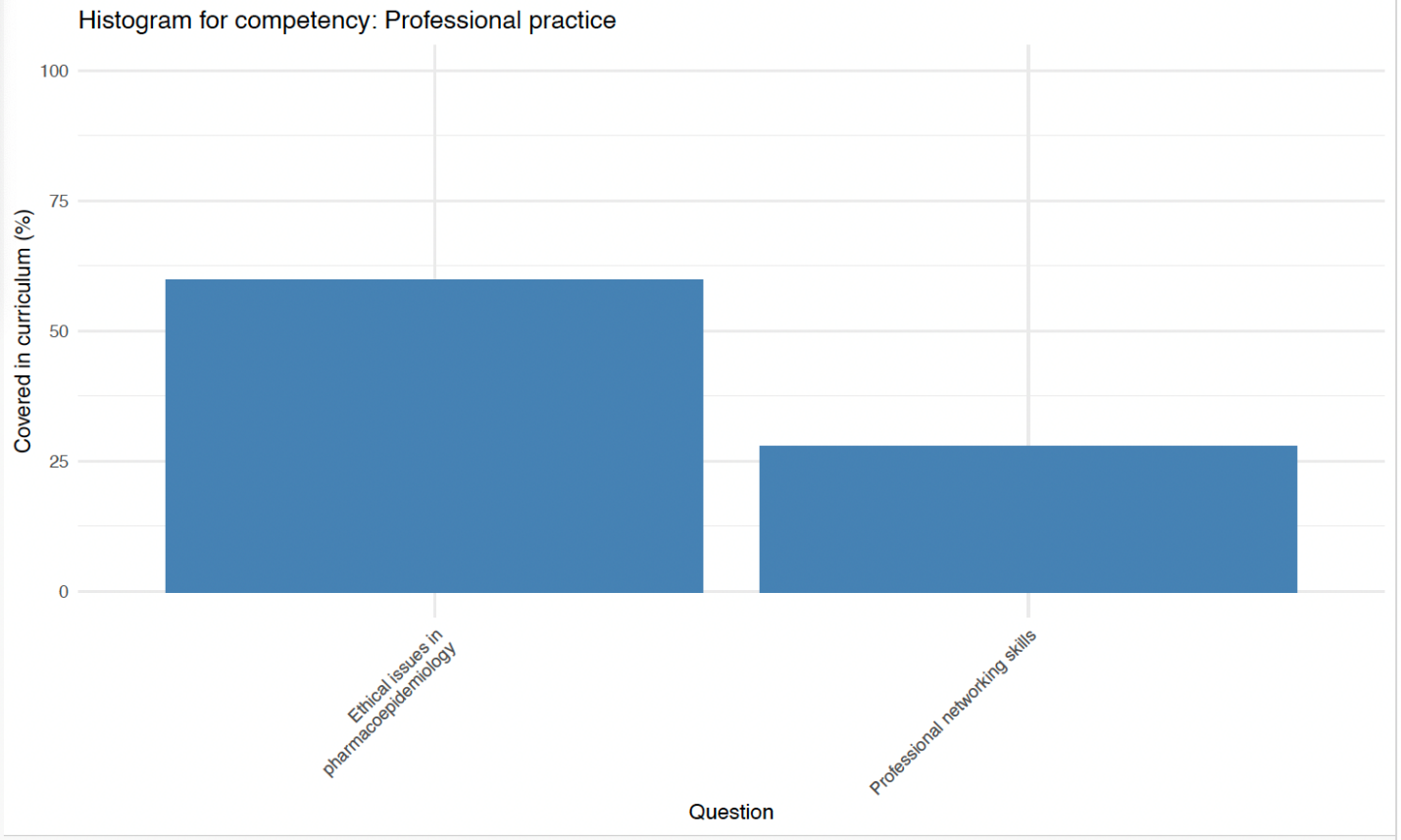


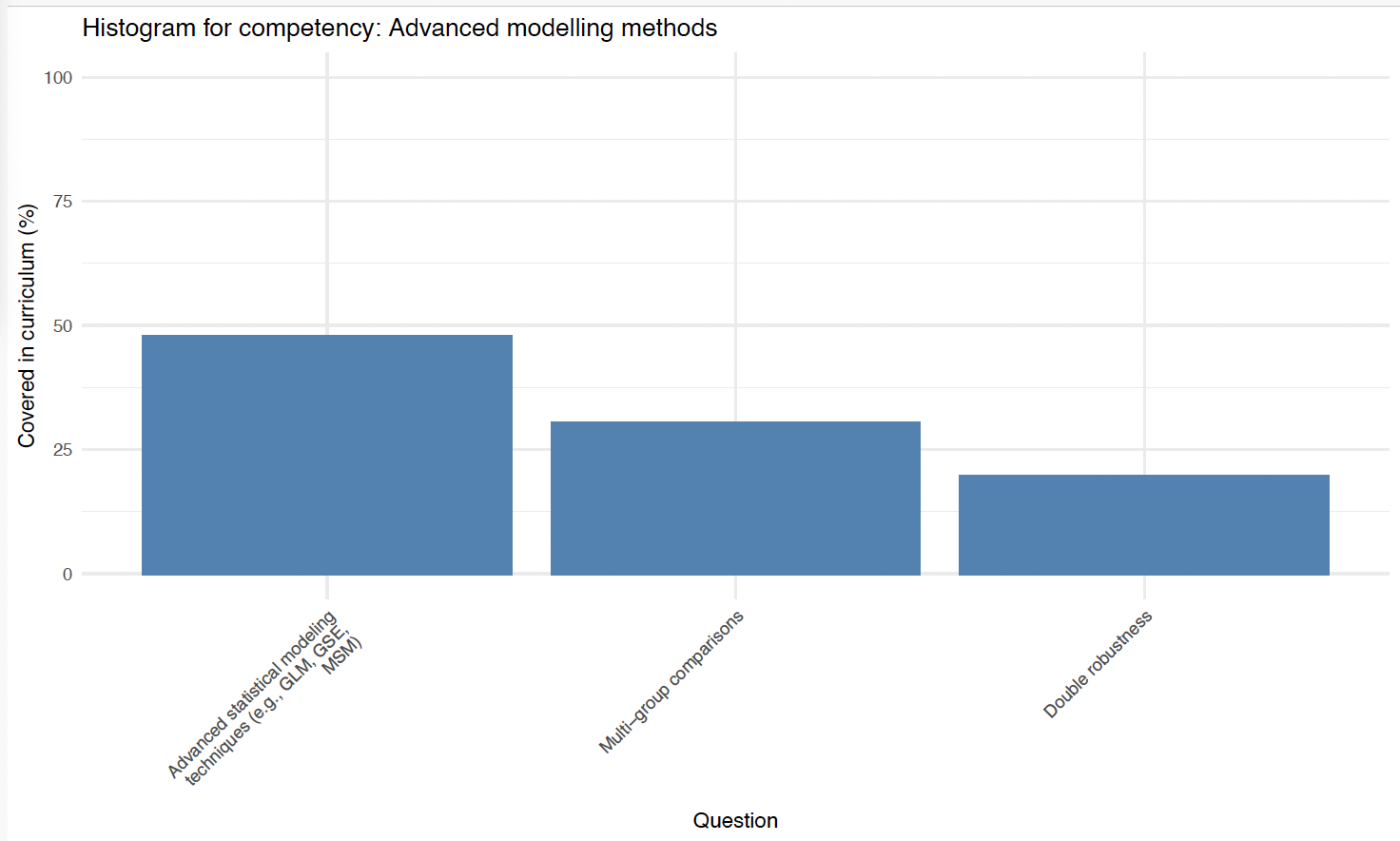

Supplement: Supplementary file 1 — Appendix 1 Pharmacoepidemiology curriculum assessment and educational needs survey instrument (attached). Appendix 2. Pharmacoepidemiology core competencies endorsed by ISPE and competencies mapped to survey items. Appendix 3. List of participating pharmacoepidemiology training programs. Appendix 4. Global representation of pharmacoepidemiology programs in the curriculum and educational needs assessment (n = 64). Appendix 5. Plots representing individual core competency coverage within educational program curriculum as organized into competency themes. [file PDS-35-e70351-s001.docx]
