# Supplementary material for: Curriculum in Pharmacoepidemiology Training Programs: A Cross‐Sectional Study to Assess Educational Needs and Alignment With Core Competencies
Source: Pharmacoepidemiol Drug Saf. 2026 Mar 26;35(4):e70351. doi: 10.1002/pds.70351 (PMC13021568; doi:10.1002/pds.70351)
Supplement: Supplementary file 2 — Data S1: Supporting Information. [file PDS-35-e70351-s002.pdf]

International Society for Pharmacoepidemiology

Program and Respondent Descriptions

Which of the following best represents the institution for which you are affiliated?

- ○ Academic
- ○ Government
- ○ Industry
- ○ Consulting
- ○ [ ] Other (please describe)

Which of the following describes your academic pharmacoepidemiology training program? (check all that apply)

- ☐ A graduate degree program in pharmacoepidemiology
- ☐ A graduate degree program that offers 1 or more pharmacoepidemiology courses
- ☐ A non-degree granting program in pharmacoepidemiology
- ☐ A non-degree granting program offering 1 or more pharmacoepidemiology courses
- ☐ [ ] Another type of educational training program (please describe)

What is your role within the academic program? (check all that apply)

- ☐ Faculty
- ☐ Administration
- ☐ [ ] Instructor or Other (please describe)

What is the name of your institution?

[ ]

What is the location (country) of your institution?

[▾]

How long have you been in pharmacoepidemiology?

- ○ <1 year
- ○ 1-2 years
- ○ 3-5 years
- ○ 6-10 years
- ○ 11-20 years
- ○ >20 years

What is your level of seniority in pharmacoepidemiology?

- ○ Currently a student or trainee
- ○ Research associate/assistant
- ○ Project management/research
- ○ Supervisory in industry/consultant
- ○ Academic associate or full professor
- ○ [ ] Other (please describe)

What types of training have you completed? (check all that apply)

- ☐ Clinical training- pharmacy
- ☐ Clinical training- medicine
- ☐ Clinical training- other health science
- ☐ Research- pharmacology
- ☐ Research- epidemiology
- ☐ Research- biostatistics
- ☐ Research- data science
- ☐ Research- other social sciences
- ☐ [ ] Other (please describe)

Pharmacoepidemiology Course Content

If courses are offered in your program, which of the following subjects are offered? (check all that apply)

- [ ] Epidemiology
- [ ] Clinical pharmacology
- [ ] Regulatory science
- [ ] Health science communication
- [ ] Statistics
- [ ] Data science
- [ ] Pharmacoepidemiology research methods

If courses are offered in your program, which of the following competencies related to advanced modeling methods are covered in one or more courses? (check all that apply)

For the purposes of this survey, we define "competencies" as the knowledge or skill required to successfully complete work in the field of pharmacoepidemiology.

- [ ] Advanced statistical modeling techniques (e.g., GLM, GSE, MSM)
- [ ] Double robustness
- [ ] Multi-group comparisons
- [ ] None of these are offered
- [ ] Not applicable

If courses are offered in your program, which of the following competencies related to statistics are covered in one or more courses? (check all that apply)

For the purposes of this survey, we define "competencies" as the

knowledge or skill required to successfully complete work in the field of pharmacoepidemiology.

- [ ] Meta-analysis
- [ ] Advanced epidemiology
- [ ] Basic/generic statistics
- [ ] Confounding and bias
- [ ] Power and sample size
- [ ] None of these are offered
- [ ] Not applicable

If courses are offered in your program, which of the following competencies related to data sources are covered in one or more courses? (check all that apply)

For the purposes of this survey, we define "competencies" as the knowledge or skill required to successfully complete work in the field of pharmacoepidemiology.

- [ ] Embedding prospective data collection in secondary databases for additional data collection
- [ ] Data sources and types of data in pharmacoepidemiology
- [ ] Quality and validation of data sources
- [ ] None of these are offered
- [ ] Not applicable

If courses are offered in your program, which of the following competencies related to interpretation of results are covered in one

or more courses? (check all that apply)

For the purposes of this survey, we define "competencies" as the knowledge or skill required to successfully complete work in the field of pharmacoepidemiology.

- ☐ Written communication of study methods, results and interpretation
- ☐ Oral presentation of study methods, results and interpretation
- ☐ Interpreting epidemiologic data: chance, bias, confounding, effect modification
- ☐ None of these are offered
- ☐ Not applicable

If courses are offered in your program, which of the following competencies related to pharmacology are covered in one or more courses? (check all that apply)

For the purposes of this survey, we define "competencies" as the knowledge or skill required to successfully complete work in the field of pharmacoepidemiology.

- ☐ Common drug associated conditions, symptoms and syndromes
- ☐ Types of adverse events (A,B) by mechanism/classification system
- ☐ Basic principles of drug actions, pharmacokinetics and pharmacodynamics
- ☐ Variability in drug response due to drug-drug interactions
- ☐ Pharmacogenomics/genetics
- ☐ None of these are offered
- ☐ Not applicable

If courses are offered in your program, which of the following competencies related to regulation are covered in one or more courses? (check all that apply)

For the purposes of this survey, we define "competencies" as the knowledge or skill required to successfully complete work in the field of pharmacoepidemiology.

- [ ] Regulatory reporting requirements
- [ ] Basic phases of drug development and information obtained
- [ ] Drug regulatory process and agencies
- [ ] Risk management
- [ ] None of these are offered
- [ ] Not applicable

If courses are offered in your program, which of the following competencies related to study design and clinical trials are covered in one or more courses? (check all that apply)

For the purposes of this survey, we define "competencies" as the knowledge or skill required to successfully complete work in the field of pharmacoepidemiology.

- [ ] Basic epidemiology study designs and their strengths/limitations
- [ ] Study designs for vaccines and other special therapeutic categories (e.g. gene therapy)
- [ ] Fundamental principals of comparative clinical trials, key decisions of design, delivery and assessment, reporting and meta-analysis
- [ ] None of these are offered

- [ ] Not applicable

If courses are offered in your program, which of the following competencies related to other quantitative analysis skills are covered in one or more courses? (check all that apply)

For the purposes of this survey, we define "competencies" as the knowledge or skill required to successfully complete work in the field of pharmacoepidemiology.

- [ ] Benefit-Risk assessment methods
- [ ] Causal mediation analysis
- [ ] Distributed data networks and use of Common Data Models
- [ ] Data mining techniques
- [ ] Demographic analysis
- [ ] Health economics modeling approaches
- [ ] Machine learning techniques
- [ ] Missing data and data imputation
- [ ] Statistical programming skills
- [ ] Quantitative bias analysis
- [ ] Interpret, design, and appraise sensitivity analysis
- [ ] None of these are offered
- [ ] Not applicable

If courses are offered in your program, which of the following competencies related to other research skills and applications are covered in one or more courses? (check all that apply)

For the purposes of this survey, we define "competencies" as the knowledge or skill required to successfully complete work in the field of pharmacoepidemiology.

- [ ] Appraisal of pharmacoepidemiological research
- [ ] Policy, public health and regulatory decision making
- [ ] Good Pharmacoepidemiology Practices guidelines
- [ ] Applications of omics data in epidemiology and public health
- [ ] Qualitative methods in health research
- [ ] Basic research skills (generic)
- [ ] Spontaneous report methods and interpretation
- [ ] Signal detection definitions and methods
- [ ] Signal evaluation approaches
- [ ] Basic principles of digital health
- [ ] Survey methodology in health research
- [ ] Measurement of exposure, outcomes and covariates
- [ ] None of these are offered
- [ ] Not applicable

If courses are offered in your program, which of the following competencies related to drug utilization or disease prevention are covered in one or more courses? (check all that apply)

For the purposes of this survey, we define "competencies" as the knowledge or skill required to successfully complete work in the field of pharmacoepidemiology.

- [ ] Global burden of communicable and noncommunicable disease and public health intervention strategies
- [ ] Disease prevention strategies (including screening)
- [ ] Drug utilization, adherence, and switching

- [ ] Geriatrics, pediatric, pregnancy and other specific and special populations
- [ ] None of these are offered
- [ ] Not applicable

If courses are offered in your program, which of the following competencies related to professional practice are covered in one or more courses? (check all that apply)

For the purposes of this survey, we define "competencies" as the knowledge or skill required to successfully complete work in the field of pharmacoepidemiology.

- [ ] Ethical issues in pharmacoepidemiology
- [ ] Professional networking skills
- [ ] None of these are offered
- [ ] Not applicable

Perceptions of Core Competencies

Which of the following competencies would you prioritize for developing standards for pharmacoepidemiology curriculum?

For the purposes of this survey, we define "competencies" as the knowledge or skill required to successfully complete work in the field of pharmacoepidemiology.

- ○ Epidemiology

- ○ Clinical pharmacology
- ○ Regulatory science
- ○ Communication and other professional skills
- ○ Statistics, analysis, and data science
- ○ [ ] Other (please describe)

What would you describe as a key need in pharmacoepidemiology curriculum (i.e., type of course content, type of method)?

[ ]

What subject area do you find most difficult to locate high quality trainings, tutorials, or other educational materials that are suitable for use in courses?

- ○ Epidemiology
- ○ Clinical pharmacology
- ○ Regulatory science
- ○ Communication and other professional skills
- ○ Statistics, analysis, and data science
- ○ [ ] Other (please describe)

What types of content would best support curriculum development

for your institution?

- ○ Example syllabi
- ○ Reading lists
- ○ Recorded webinars/lectures
- ○ Other standardized materials (i.e., learning objectives with pre-packaged activities)
- ○ [ ] Other (please describe)

Powered by Qualtrics
